# Maternal Religiosity and Adolescent Substance Use: A UK Prospective Cohort Study

**DOI:** 10.1007/s10943-025-02299-2

**Published:** 2025-04-06

**Authors:** Isaac Halstead, Jon Heron, Carol Joinson

**Affiliations:** 1 The Centre for Academic Child Health, Population Health Sciences, Bristol Medical School, University of Bristol, Bristol, BS8 2BN UK; 2 Population Health Sciences, Bristol Medical School, University of Bristol, Bristol, BS8 2BN UK

**Keywords:** ALSPAC, Religion, Substance use, Latent class analysis, Religiosity, Spirituality

## Abstract

Adolescent substance use can have a significant negative impact on life trajectories. Therefore, identifying factors associated with adolescent substance use is important. Previous research has identified parental religiosity as a factor associated with lower adolescent substance use. However, these studies suffered from a number of limitations and are often focussed on US samples, which limit the generalisability of their findings. The present study used a large UK-based longitudinal cohort study (*n* = 8041) and latent classes of parental religious belief at age 9 to examine the association with offspring adolescent substance use at age 18, while controlling for a range of confounders. We found evidence that suggests, when compared to offspring of agnostic mothers, having a highly religious or atheist mother is associated with lower odds of offspring weekly smoking (OR 0.68 [0.45, 1.02] and OR 0.74 [0.53, 1.04] respectively), and having an atheist mother is associated with greater odds of cannabis (OR 1.32 [1.05, 1.66]) and other drugs use (OR 1.41 [1.02, 1.95]). Our findings suggest that parental beliefs can have an impact on adolescent outcomes, and these associations may be generalisable to non-US contexts.

## Introduction

Adolescence is a developmentally sensitive period, when individuals are more likely to engage in risky behaviours, such as substance use (e.g. alcohol, illegal drugs, tobacco) (Hall et al., 2016). Substance use during adolescence can have a significant impact on life trajectories and is associated with adverse outcomes including mental health problems, involvement in crime, poor educational attainment, and adult substance misuse) (Englund et al., 2013; Lynne-Landsman et al., 2010; Taylor et al., 2017). Factors associated with an increased risk of adolescent substance use include a higher level of sensation seeking (Quinn & Harden, 2013), parental substance use (Yule et al., 2018), low parental monitoring (Rusby et al., 2018), low socioeconomic position (SEP) (Karriker-Jaffe, 2011; Patrick et al., 2012), and conduct problems (Heron et al., 2013).

There is also some evidence that parental religiosity plays a role in young people’s substance use. Parental religiosity has been found to be a protective factor against substance use in several studies (Hayatbakhsh et al., 2014; Hoffmann, 2023; Jankowski & Zamboanga, 2019; Mak, 2018) but one other study found no association (Parsai et al., 2010). There are limitations in the previous literature that limit understanding of the association between religiosity and substance use. The studies often rely upon single item measures of religious belief, such as church attendance or the importance of religion in the parent’s life. These measures are unable to differentiate between those who have no religious belief, weak religious belief, or are who agnostic. Single items cannot capture the multifaceted nature of religious belief and could distort differential associations with substance use. For example, previous studies found that religious importance has a weaker association with substance use than church attendance (e.g. Kelly et al., 2015; Livne et al., 2021). These limitations suggest using methods that are able to differentiate between qualitatively different groups of religious (non)belief, such as latent class analysis in the present study.

Much of the earlier research is conducted on US samples (e.g. Foshee & Hollinger, 1996; Hoffmann, 2023; Kim-Spoon et al., 2014; Merrill et al., 2005). The US has a relatively high level of belief in God (approximately 82%; Duffy et al., 2023) compared to other countries such as the UK (approximately 49% (Duffy et al., 2023)). Furthermore, even within those who may have a belief in god in the UK, they may not necessarily identify with a religion (Woodhead, 2017), or being religious (Oake, 2021). There is evidence of more benefits to the individual (e.g. social support, inclusion, lower likelihood of prejudice Chuah et al., 2016; Gebauer & Sedikides, 2021; Ng & Gervais, 2016; Stavrova & Siegers, 2014)) associated with being religious within a religious majority social group, compared with being non-religious in a religious majority social group. Some examples of differences in US and UK samples can be seen in recent work examining mental health outcomes, which found that being religious was a risk factor for some offspring mental health outcomes, such as self-harm, oppositional defiant disorder, and depression (Halstead et al., 2023, 2024), which is the opposite of the majority of the US literature. It is possible that between country differences may extend to the association between religious belief and substance use. Therefore, we have chosen to examine a UK sample.

Many studies in religious belief research obtain their samples opportunistically, often in university student populations (Koenig, 2012), and examine cross-sectional associations (Kelly et al., 2015). The use of opportunistic sampling in university populations student (i.e. younger, better educated participants) can introduce selection bias based on SEP factors (Howe et al., 2013; Munafò et al., 2018), which are predictive of substance use (Heron et al., 2013). Reverse causality is possible in cross-sectional studies, which has been observed previously in the case of criminal behaviour predicting religion (Heaton, 2006). In the context of the present study, offspring substance use may introduce issues to the family environment (such as navigating possible drug dependency, changes in behaviour due to substance use), which may impact a parent’s religious belief, or ability to practice it. Furthermore, we intend to address the issue of the use of student populations by examining a sample taken from the general population.

The current study, based on data from a large birth cohort, examines prospective associations between different patterns of maternal religious belief, and adolescent offspring substance use (alcohol, cigarette smoking, cannabis use, other drugs use, such as amphetamines, heroin, and cocaine) at age 17.5 years. We seek to examine whether maternal religious (non) belief is associated with subsequent substance use by their offspring, when controlling for confounders and examined longitudinally.

## Methods

### Participants

The Avon Longitudinal Study of Parents and Children (ALSPAC) was established to understand how genetic and environmental characteristics influence health and development in parents and children. All pregnant women resident in a defined area (the Avon county and Bristol area) in the Southwest of England, with an expected date of delivery between 1st April, 1991 and 31st December, 1992 were invited to take part in the study. The initial number of pregnancies enrolled is 14,541. Of these initial pregnancies, there were a total of 14,676 foetuses, resulting in 14,062 live births and 13,988 offspring who were alive at 1 year of age. When the oldest children were approximately 7 years of age, an attempt was made to bolster the initial sample with eligible cases who had failed to join the study originally. As a result, when considering variables collected from the age of seven onwards, there are data available for more than the 14,541 pregnancies. These parents and offspring have been followed over the last 30 years and have completed a variety of questionnaires concerning their demographics, physiological and genetic data, life events, physical, and psychological characteristics. For more information, see (Boyd et al., 2013; Fraser et al., 2013). The study website contains details of all data that is available through a fully searchable data dictionary and variable search tool (http://www.bristol.ac.uk/alspac/researchers/our-data/).

## Exposures

### Latent Classes of Maternal Religiosity

Latent class analysis is a ‘person-centred’ statistical technique (Nylund-Gibson & Choi, 2018) that uses a set of observed variables and the conditional probability of responding in a particular pattern to those variables to probabilistically assign participants to a mutually exclusive unobserved group (i.e. latent class). We have previously derived maternal latent class memberships as indicators of religiosity in the mothers of the ALSPAC parent cohort (Halstead et al., 2022, 2023, 2024), based upon the following assumptions: mothers are the primary caregivers, the classes are stable over time (D. Smith et al., 2022), maternal and paternal religious latent classes are strongly associated^22^. In the current study, we used maternal religiosity assessed when the study child was age 9 years, because these were the most proximal variables to the adolescent substance use outcomes.

We labelled the classes the highly religious, moderately religious, agnostic, and atheist and each represented approximately 19%, 24%, 38%, and 19% of the sample, respectively. The highly religious exhibit a consistent endorsement of religious belief, high probability of regular attendance at a place of worship, and high likelihood to obtain help and support from religious individuals. The moderately religious share many of the characteristics of the highly religious but were very unlikely to attend a place of worship regularly. The agnostic expressed uncertainty about the existence of God and was also uncertain about whether they would ask for help from God if they were in trouble or whether God had helped them previously. The atheist expressed strong disbelief in the existence of God, and general disagreement with statements related to religious belief and practice. For details of the questions used to derive the latent classes and the conditional probabilities of each class, see Appendices 1 and 3, respectively.

## Outcomes

The data were collected as part of the ‘Teen focus 4’ clinic, when the offspring were approximately 17.5 years old (mean = 17.82, SD = 0.456).

### Alcohol Use

Respondents completed the ten-item alcohol use disorders identification test (AUDIT) (World Health Organization et al., 2001). We used a cut-off of 16 points and above on the AUDIT scale to indicate harmful use.

### Smoking

Following positive responses to ‘‘having ever smoked’’ and ‘‘having smoked in the last 30 days’’, respondents indicated whether they smoked every week. The response to this question was used as binary variable.

### Cannabis Use

Respondents completed the six-item cannabis abuse screen test (CAST) (Legleye et al., 2013) asking about cannabis use in the previous 12 months. The sum score was derived by assigning 1 to the responses ‘‘fairly often’’ and ‘‘often’’ and 0 to the other response options and summing the responses. This scale was then dichotomized to indicate those scoring one or more points. We opted for this cut-off to yield an adequate number of problem users.

### Illicit Drugs

A series of questions was asked about the use of cocaine, amphetamines, inhalants, sedatives, hallucinogens or opioids in the previous 12 months. Respondents were assigned a score of 1 if they reported having used any of the drugs listed.

## Confounders

Analyses were adjusted for the potential confounding effects of parental occupational social class, maternal age, maternal educational attainment, economic hardship/problems, maternal anxiety and depression, maternal adverse childhood events (reported retrospectively) and child sex as assigned at birth. See Appendix 2 for more information.

### Statistical Analyses

We used multivariable logistic regression to calculate odds ratios and 95% confidence intervals for the association between the maternal religious latent classes and each substance use outcome. Odds ratios were estimated in relation to the agnostic class, as this was the largest class in the sample and was characterised by the most ‘neutral’ beliefs (i.e. neither religious nor Atheist) (Johfre & Freese, 2021). Parameter estimates were then adjusted for confounders. The dataset was constructed in R studio (R Core Team, 2021), and all analyses were carried out in Mplus (Version 8.7), using a bias adjusted 3-step latent class analysis which incorporates uncertainty in latent class assignment (Vermunt & Magidson, 2021). The analysis was not pre-registered, and the results should be considered exploratory.

### Missing Data

We conducted the primary analysis on an imputed dataset of 8041 mothers and offspring who had maternal religiosity measures. We used multivariate imputation by chained equations to impute missing data on the substance use outcomes and confounders using the *mice* package in R (Buuren & Groothuis-Oudshoorn, 2011) under the missing at random (MAR) assumption. In addition to variables in the main analysis, we included auxiliary variables that were likely to be related to the missing data in the imputation model. These included previous measures of drinking, smoking, cannabis use and whether they had been offered drugs at age 13.5. We used *mice* to impute 100 datasets with 100 iterations per dataset which were used in Mplus for analysis, which provided averaged model estimates across the datasets. While there is not a well-established technique for addressing missing covariate data in LCA, the approach in the current paper has been used previously (Kretschmer et al., 2014). For information on missingness in the complete sample, see Appendix 4. Our approach to multiple imputation was informed by recommendations from the literature (Sterne et al., 2009; White et al., 2011).

**Fig. 1:**
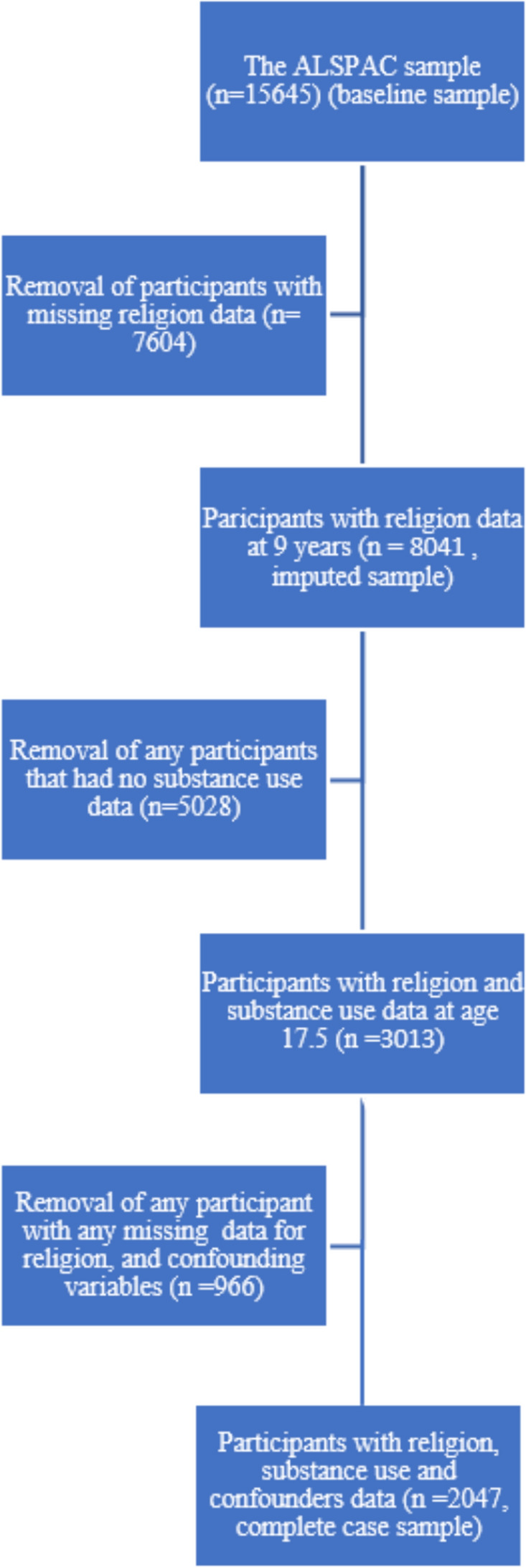
Study sample flowchart for both the imputed and complete case samples

We also conducted the analysis on the sample with complete data (2047 mothers and offspring with complete religiosity, substance use outcomes and confounders). We report these results in the Appendices. See Appendix 5 for the results of these complete case analyses (see Fig. 1 for details of the removals and inclusions).

## Results

In comparison to the complete case sample, the imputed sample had higher proportions of alcohol use, cannabis use, drug use and smoking. They also had higher proportions of low SEP indicators (social class, education, financial problems/hardship) (See Table 1 for more information).

**Table 1:** Distribution of variables across the complete case and imputed datasets for substance use outcomes

Variable	Reference	Complete case sample (*n* = 2047)		Imputed sample (*n* = 8041)	
		Median (IQR)	N(%)	Median (IQR)	N(%)
Harmful alcohol use	Non-harmful drinking (harmful drinking)		77(3.8%)		548(6.8%)
Weekly smoking	Not weekly smoker (weekly smoker)		226 (11.0%)		983 (12.2%)
Cannabis	Not used (used)		562 (27.5%)		2406 (29.9%)
Other drugs	Not used (used)		217 (10.6%)		1067 (13.3%)
Maternal crime	No criminal record (a criminal record)		91 (4.4%)		507 (6.3%)
Maternal substance misuse	No substance misuse (misused substances)		386 (18.9%)		1543 (19.2%)
Maternal smoking	Smoker (non-smoker)		232 (11.3%)		1605 (20.0%)
Sex assigned at birth	Male (Female)		1105 (54.0%)		3966 (49.3%)
Maternal education	A level or higher (No education/CSE/vocational; GCSE)		253 (12.4%) 676 (33.0%)		1800 (22.4%) 2846 (35.4%)
Social class	Non-manual (manual)		131 (6.4%)		821 (10.2%)
Home status	Owned (rented)		221 (10.8%)		1536 (19.2%)
Financial problems	No problems (had problems)		168 (8.2%)		875 (10.9%)
Financial hardship	No difficulties (had difficulties)		312 (15.2%)		1796 (22.3%)
Maternal age at birth of study child		30 (6)		29 (6)	
Marital status	Not single (single)		254 (12.4%)		1535 (19.1%)
Maternal anxiety		4 (4)		4 (5)	
Maternal depression		5 (5)		6 (6)	
Maternal stressful life events		3 (3)		3 (3)	

In the complete case sample, compared to offspring of agnostic mothers, offspring of atheist mothers had higher odds of cannabis use and other drugs use [OR 1.33(1.01, 1.75) and OR (1.68 (1.12, 2.51), respectively], but these associations were attenuated in the fully adjusted model [OR 1.16 (0.87, 1.55) and OR 1.50 (0.99, 2.27), respectively]. There was also weak evidence of an association between offspring of highly religious mothers and higher odds of other drug use [OR 1.47(0.94, 2.30)] and lower odds of smoking weekly [OR 0.60 (0.36, 1.00)].

See Table 2 for the results of the models using imputed data in the imputed sample, compared to the offspring of the agnostic mothers, the offspring of atheist mothers had greater odds of cannabis and other drug use [OR 1.32 (1.05, 1.66) and OR 1.41 (1.02, 1.95), respectively]. There was also weak evidence of offspring of highly religious mothers and offspring of atheist mothers having lower odds of smoking [OR 0.68 (0.45, 1.02) and [OR 0.72 (0.50, 1.04), respectively].

**Table 2:** Odds ratios and 95% confidence intervals for the association between the maternal religiosity latent classes and substance use outcomes in adolescent offspring at age 18 years (*n* = 8041)

	Unadjusted model		Fully adjusted model	
	OR (95% CIs)	P-value	OR (95% CIs)	P-value
**Harmful alcohol use**		0.238		0.547
Agnostic (REF)	1.00		1.00	
Atheist	0.96 (0.61,1.53)		0.89 (0.56,1.40)	
Moderately religious	0.53 (0.27,1.00)		0.91 (0.57,1.44)	
Highly religious	0.86 (0.57,1.30)		0.65 (0.36,1.17)	
**Weekly smoking**		0.125		0.126
Agnostic (REF)	1.00		1.00	
Atheist	0.77 (0.55, 1.07)		0.74 (0.53, 1.04)	
Moderately religious	1.01 (0.77, 1.33)		1.00 (0.76, 1.31)	
Highly religious	0.68 (0.45, 1.02)		0.68 (0.45, 1.02)	
**Cannabis**		0.001		0.023
Agnostic (REF)	1.00		1.00	
Atheist	1.44 (1.16, 1.81)		1.32 (1.05, 1.66)	
Moderately religious	0.94 (0.77, 1.15)		0.93 (0.75, 1.14)	
Highly religious	1.01 (0.78, 1.30)		1.00 (0.76, 1.32)	
**Other drugs**		0.028		0.151
Agnostic (REF)	1.00		1.00	
Atheist	1.55 (1.14, 2.11)		1.41 (1.02, 1.95)	
Moderately religious	1.01 (0.75, 1.36)		1.00 (0.74, 1.35)	
Highly religious	1.19 (0.85, 1.68)		1.19 (0.83, 1.69)	

## Discussion

We found evidence in a large UK birth cohort sample that, compared to the offspring of agnostic mothers, offspring of atheist mothers were more likely to have smoked cannabis or tried other drugs, and the offspring of highly religious mothers were less likely to smoke weekly. These associations were robust to the inclusion of maternal SEP indicators, maternal mental health, previous substance use, and stressful life events and were demonstrated within both the complete case and the imputed analyses.

## Comparisons with Previous Research

Our findings align with most of the studies that examined the association between religious belief and adolescent substance use in both the UK and US, with greater religious belief associated with lowers odds of smoking (Hussain et al., 2019; Nunziata & Toffolutti, 2019; Whooley et al., 2002) and lower religious belief associated with greater odds of substance use (King et al., 2013; Moscati & Mezuk, 2014). However, these studies examined an individual’s own religious belief, rather than the impact of their parent’s religious belief. In adolescence, offspring and parental religious belief may be very similar (Leonard et al., 2013), and the associations we see in the present study may be attributable to this similarity. Future studies with both measures of parental and offspring religious belief available to them may be able to explore this possibility.

## Possible Mechanisms

Higher levels of parental monitoring and control by religious parents (Kim & Wilcox, 2014; Kim-Spoon et al., 2014) may lead to offspring being less likely to try cigarettes, for fear of detection. Religious parents may be less likely to use substances themselves, so their offspring may follow the example set by their parents and avoid drugs (Kendler et al., 2003, 2015). Lower levels of parental control and monitoring (Kim & Wilcox, 2014), as well as a more open-minded (Gurney et al., 2013), less traditionalist attitude (Saroglou, 2010; Saroglou et al., 2004) may lead to offspring of atheist parents feeling comfortable with trying cannabis or other drugs. The moral views of parents may also be passed on to their offspring, informing their decision-making (Chen et al., 2024), which in the case of children of religious parents may include abstinence related decisions.

There is also the possibility that social factors may explain the associations (Ford & Hill, 2012; C. Smith, 2003). Offspring of religious parents may be more likely to have a religious social group (Stroope, 2012) and community services which may emphasise ‘Christian’ values such as abstinence (Rosansky & Rosenberg, 2019), whereas the offspring of atheist parents may rely upon secular sources of community and socialising, which may not have the same emphasis on abstinence as religious groups. Given that much substance use is based on peer influence from those engaged in risky behaviours (Allen et al., 2012; Henneberger et al., 2021), being less exposed to these influences may reduce the risk to offspring of religious parents.

## Strengths and Limitations

The use of latent class analysis allowed us to capture qualitatively different types of belief, rather than relying on single item measures. The latent classes differentiated between highly and moderately religious classes as well as atheist and agnostic classes. We used multiple imputation to address bias due to missing data, as seen in the complete case sample, where the group analysed had higher prevalences of measures of low SEP than the original cohort.

However, there are limitations of the present study that should be considered when interpreting the findings. The ALSPAC cohort is predominantly White, and those who identify as religious are mostly affiliated with Christianity. This means that the current results may not generalise to non-white, non-Christian religious populations. The study is also limited in making inferences about the time point in which the data was collected (1990–2007). The relationship that young people in the UK have with substance use has changed over time, notably experiencing a significant drop in substance use (based on lower numbers of young people accessing treatment services) (Young People’s Substance Misuse Treatment Statistics 2021 to 2022, 2023), In addition, the proportion of the population who believe in God (Duffy et al., 2023) has changed since the data were collected (e.g. 62% of people believed in God in 1999, when the religion data were collected, compared to approximately 49% in 2021). Whether the associations found in this study hold in the present day would be a valuable exploration. Finally, we did not have access to measures of the offspring’s own religious belief. Future research should examine the role of the young person’s own religious belief, which may provide an insight into the mechanisms between parental religious beliefs and offspring substance use.

## Conclusions

We found, when compared to the offspring of agnostic mothers, the offspring of atheist mothers had higher odds of using cannabis or trying other drugs, and the offspring of highly religious mothers was less likely to smoke weekly. These results provide a novel insight into the role of parental religiosity in adolescent substance use in an understudied population, using methods that overcome limitations of the previous literature. Future work should explore the role of parental religious belief independently of offspring religious belief, as well as exploring mechanisms that explain the association, such as parenting variables and social groups.

## Data Availability

Please see the ALSPAC data management plan which describes the policy regarding data sharing (http://www.bristol.ac.uk/alspac/researchers/data-access/documents/alspac-data-management-plan.pdf), which is by a system of managed open access. Data used for this submission will be made available on request to the Executive (alspac-exec@bristol.ac.uk). The datasets presented in this article are linked to ALSPAC project number B4512, please quote this project number during your application. The steps below highlight how to apply for access to the data included in this study and all other ALSPAC data: 1. Please read the ALSPAC access policy (http://www.bristol.ac.uk/media-library/sites/alspac/documents/researchers/data-access/ALSPAC_Access_Policy.pdf) which describes the process of accessing the data and samples in detail and outlines the costs associated with doing so. 2. You may also find it useful to browse our fully searchable research proposals database (https://proposals.epi.bristol.ac.uk/?q=proposalSummaries), which lists all research projects that have been approved since April 2011. 3. Please submit your research proposal (https://proposals.epi.bristol.ac.uk/) for consideration by the ALSPAC Executive Committee. You will receive a response within 10 working days to advise you whether your proposal has been approved.

## Appendix 1 Original and recoded response options for religiosity items

**Table:**

Measure	Original response options	Recoded response options	Frequency
1) Do you believe in God or in some divine power?	Yes	Yes	49.8%
	Not sure	Not sure	35.3%
	No	No	14.9%
2) Do you feel that God (or some divine power) has helped you at any time?	Yes	Yes	33.9%
	Not sure	Not sure	37.9%
	No	No	28.3%
3) Would you appeal to God for help if you were in trouble?	Yes	Yes	46.6%
	Not sure	Not sure	31.4%
	No	No	22.1%
4) How long have you had this particular faith?	All my life^1^	Long-time (non) believer^1^	96.9%
	More than 5 years^1^		
	3–5 years^2^	Recent convert^2^	3.1%
	1–2 years^2^		
	Less than a year^2^ No response^1^		
5) Do you go to a place of worship?	Yes, at least once a week^1^	Religious attendance^1^	14.3%
	Yes, at least once a month^1^	Occasional attendance^2^	85.7%
	Yes, at least once a year^2^		
	Not at all^2^		
	No response^2^		
6) Do you obtain help and support from leaders [other members] of your [other] religious group?	Yes^1^	Yes^1^	11.3%
	No^2^	No^2^	88.7%
	No response^2^		

Superscript indicates recoding pattern.

## Appendix 2 Details of variables used in analysis

**Table:**

Alcohol use	FJAL1000, FJAL1050, FJAL1100, FJAL1150, FJAL1350, FJAL1400, FJAL1450, FJAL1550, FJAL1900, FJAL1950	Frequency YP has a drink containing alcohol	Scores of 8 or more as an indicator of hazardous/harmful drinking	17.5 years
		Number of drinks containing alcohol YP has on a typical day		
		Frequency YP has 6 or more drinks on one occasion		
		Frequency YP was not able to stop drinking once started, in past year		
		Frequency YP was unable to do what was normally expected of them due to drinking, in past year		
		Frequency YP has needed a first drink in the morning to get them going after a heavy drinking session, in past year		
		Frequency YP has had a feeling of guilt or remorse after drinking, in past year		
		Frequency YP has been unable to remember what happened the night before because they had been drinking, in past year		
		YP or someone else has been injured as a result of YPs drinking		
		Relative or friend or doctor or other health worker has been concerned about YPs drinking or suggested they cut down		
Smoking	FJSM050	YP has ever smoked a whole cigarette	Any weekly smoking	17.5 years
	FJSM250	YP has smoked cigarettes in the past 30 days		
	FJSM450	(SMOKE EVERY WEEK)		
Cannabis use	FJDR1000, FJDR1050, FJDR1100, FJDR1150, FJDR1200, FJDR1250	YP has ever used cannabis before midday, in the past 12 months	A score greater than 1	17.5 years
		YP has ever used cannabis when they were alone, in the past 12 months		
		YP has ever had memory problems when they used cannabis, in the past 12 months		
		YP has friends or family members tell them they ought to reduce their cannabis use, in the past 12 months		
		YP has ever tried to reduce or stop their cannabis use without succeeding, in the past 12 months		
		YP has ever had problems because of their use of cannabis (argument/fight/accident/bad result at school etc.), in the past 12 months		
Illicit drugs	FJDR5050	YP has tried cocaine in the last year	Any drug use	17.5 years
	FJDR5200	YP has tried amphetamines in the last year		
	FJDR5350	YP has tried inhalants in the last year		
	FJDR5500	YP has tried Sedatives or Sleeping Pills in the last year		
	FJDR5650	YP has tried Hallucinogens in the last year		
	FJDR5800	YP has tried Opioids in the last year		
	FJDR5950	YP has tried other substance in the last year		
*Covariates/confounders*				
Occupational social class	C755/C765	The highest of paternal/maternal occupational social class was used	Manual/non-manual	Antenatal
Maternal age at birth	e695	Age of mother at birth		Antenatal
Maternal education	c645a	Mums highest ed qualification	CSE/none/Vocational	Antenatal
			O level	
			A level/Degree	
Economic hardships/problems	b594	Major financial PROB since PREG	Yes/No	Antenatal
	c525	Financial difficulties (scores greater than 6 on the financial difficulties scale were coded as experiencing difficulties)	Scores above 6 were taken as a sign of financial difficulties	
Maternal drug/alcohol use		FAI drug sub scores – substance use in the first 4 years after pregnancy	0–4 years	Antenatal-4 years
Maternal Smoking	j735	Number of cigarettes smoked a day	Yes/No	4 years
Child sex assigned at birth	KZ021		Male/Female	At birth
Marital status	a525	PRES marital status	Married/Single	Antenatal
Housing status	g352	Home rented or owned	Rented/owned	1.5 years old
Maternal stressful life events	e445	Total stressful life events		Antenatal
Maternal depression	b370	EPDS depression score		Antenatal
Maternal anxiety	B351	Crown-crisp anxiety score		Antenatal
*Auxiliary variables*				
Smoked cigarettes	fg4822	Teenager has smoked cigarettes	Yes/No	13.5 years old
Alcohol use	fg4872	Teenager has drunk alcohol without parents’ permission	Yes/No	13.5 years old
Cannabis use	fg5422	Teenager has tried cannabis	Yes/No	13.5 years old
Offered drugs	fg5500	Teenager has been offered drugs	Yes/No	13.5 years old

## Appendix 3 Conditional probabilities for each maternal latent class

**Table:**

	Mothers			
	Highly religious:	Moderately religious:	Agnostic:	Atheist:
*Do you believe in God or in some divine power?*				
Yes	1.00	0.93	0.24	0.06
Not sure	0.00	0.07	0.75	0.24
No	0.00	0.00	0.01	0.70
*Do you feel that God (or some divine power) has helped you at any time? *				
Yes	0.95	0.73	0.02	0.00
Not sure	0.05	0.25	0.78	0.01
No	0.00	0.02	0.20	0.99
*Would you appeal to God for help if you were in trouble? *				
Yes	0.98	0.89	0.22	0.01
Not sure	0.01	0.09	0.71	0.08
No	0.00	0.01	0.08	0.91
*How long have you had this particular faith? *				
Long-time (non)believer	0.89	0.96	1.00	0.97
Recent convert	0.11	0.04	0.00	0.03
*Do you go to a place of worship? *				
Religious attendance	0.87	0.09	0.03	0.01
Occasional attendance	0.13	0.91	0.97	0.99
*Do you obtain help and support from leaders or other members of your [other] Religious group? *				
Yes	0.84	0.03	0.02	0.00
No	0.16	0.97	0.98	1.00

## Appendix 4 Missingness for the pre-imputed sample

**Table:**

Variable	Imputed sample size = 8041
	Missing (%)
Alcohol	4704
	(58.5%)
Cannabis	4706
	(58.5%)
Other drugs	4728
	(58.8%)
Smoking	4546
	(56.5%)
Maternal crime	330
	(4.1%)
Maternal alcohol/drug use	357
	(4.4%)
Maternal smoking	1075
	(13.4%)
Maternal education	520
	(6.5%)
Social class	1298
	(16.1%)
Home status	919
	(11.4%)
Financial problems	1018
	(12.7%)
Financial hardship	708
	(8.8%)
Age at birth	637
	(7.9%)
Marital status	465
	(5.8%)
Maternal depression	979
	(12.2%)
Maternal anxiety	1068
	(13.3%)
Maternal stressful events	634
	(7.9%)

## Appendix 5 Odds ratios and 95% confidence intervals for the association between the maternal religiosity latent classes and substance use outcomes in adolescent offspring at age 18 years in the complete case sample (*n* = 2047)

**Table:**

Unadjusted models					Fully adjusted models				
**Alcohol**				0.457					0.543
Atheist	0.80	0.42	1.53		Atheist	0.74	0.38	1.46	
Moderately religious	0.60	0.24	1.51		Moderately religious	0.59	0.23	1.51	
Highly religious	0.58	0.26	1.29		Highly religious	1.52	0.69	3.33	
**Cannabis**				0.038					0.370
Atheist	1.33	1.01	1.75		Atheist	1.16	0.87	1.54	
Moderately religious	0.84	0.59	1.20		Moderately religious	0.85	0.60	1.21	
Highly religious	0.90	0.66	1.24		Highly religious	0.91	0.66	1.26	
**Other drugs**				0.053					0.167
Atheist	1.68	1.12	2.51		Atheist	1.50	0.99	2.27	
Moderately religious	1.28	0.78	2.11		Moderately religious	1.29	0.79	2.16	
Highly religious	1.51	0.97	2.34		Highly religious	1.44	0.94	2.30	
**Smoking**				0.231					0.230
Atheist	0.87	0.58	1.30		Atheist	0.81	0.54	1.23	
Moderately religious	0.88	0.54	1.42		Moderately religious	0.84	0.52	1.39	
Highly religious	0.60	0.36	0.98		Highly religious	0.58	0.36	1.00	
